# Predicting Postoperative Length of Stay in Patients Undergoing Laparoscopic Right Hemicolectomy for Colon Cancer: A Machine Learning Approach Using SICE (Società Italiana di Chirurgia Endoscopica) CoDIG Data

**DOI:** 10.3390/cancers16162857

**Published:** 2024-08-16

**Authors:** Gabriele Anania, Matteo Chiozza, Emma Pedarzani, Giuseppe Resta, Alberto Campagnaro, Sabrina Pedon, Giorgia Valpiani, Gianfranco Silecchia, Pietro Mascagni, Diego Cuccurullo, Rossella Reddavid, Danila Azzolina, On behalf of SICE CoDIG (ColonDx Italian Group)

**Affiliations:** 1 Department of Medical Science, University of Ferrara, 44121 Ferrara, Italy; gabriele.anania@unife.it (G.A.); g.resta@ospfe.it (G.R.); cmplrt@unife.it (A.C.); sabrina.pedon@edu.unife.it (S.P.); 2 Clinical Trial and Biostatistics, Research and Development Unit, University Hospital of Ferrara, 44121 Ferrara, Italy; emma.pedarzani@ubep.unipd.it (E.P.); giorgia.valpiani@ospfe.it (G.V.); 3 Unit of Biostatistics, Epidemiology and Public Health, Department of Cardiac Thoracic, Vascular Sciences, University of Padua, 35122 Padua, Italy; 4 Department of Scienze Medico Chirurgiche e Medicina Traslazionale, University of Roma, S. Andrea University Hospital, 00189 Rome, Italy; gianfranco.silecchia@uniromai.it; 5 Fondazione Policlinico Universitario Agostino Gemelli IRCCS, 00136 Rome, Italy; 6 Institute of Image-Guided Surgery, IHU-Strasbourg, 67000 Strasbourg, France; pietro.mascagni@ihu-strasbourg.eu; 7 Division of Laparoscopic and Robotic Surgery Unit, A.O.R.N. dei Colli Monaldi Hospital, 80131 Naples, Italy; diego.cuccurullo@ospedalideicolli.it; 8 Division of Surgical Oncology and Digestive Surgery, Department of Oncology, San Luigi University Hospital, University of Turin, Orbassano, 10043 Turin, Italy; rossella.reddavid@unito.it; 9 Department of Preventive and Environmental Science, University of Ferrara, 44121 Ferrara, Italy; danila.azzolina@unife.it

**Keywords:** machine learning, right hemicolectomy, colon cancer, laparoscopy, length of stay

## Abstract

**Simple Summary:**

This study aimed to predict the Length of hospital Stay (LoS) after laparoscopic right hemicolectomy for colon cancer using machine learning techniques. Accurately forecasting LoS is crucial for improving patient care and hospital resource management. The researchers utilized data from two large Italian studies, CoDIG 1 and CoDIG 2, to train and validate various machine learning models. The Random Forest (RF) algorithm demonstrated the best internal performance, while the Support Vector Machine (SVM) outperformed in external validation. Key factors influencing LoS included the use of fast-track protocols, type of anastomosis, and drainage. These findings could help tailor postoperative care and optimize hospital resources, ultimately enhancing patient outcomes and operational efficiency.

**Abstract:**

The evolution of laparoscopic right hemicolectomy, particularly with complete mesocolic excision (CME) and central vascular ligation (CVL), represents a significant advancement in colon cancer surgery. The CoDIG 1 and CoDIG 2 studies highlighted Italy’s progressive approach, providing useful findings for optimizing patient outcomes and procedural efficiency. Within this context, accurately predicting postoperative length of stay (LoS) is crucial for improving resource allocation and patient care, yet its determination through machine learning techniques (MLTs) remains underexplored. This study aimed to harness MLTs to forecast the LoS for patients undergoing right hemicolectomy for colon cancer, using data from the CoDIG 1 (1224 patients) and CoDIG 2 (788 patients) studies. Multiple MLT algorithms, including random forest (RF) and support vector machine (SVM), were trained to predict LoS, with CoDIG 1 data used for internal validation and CoDIG 2 data for external validation. The RF algorithm showed a strong internal validation performance, achieving the best performances and a 0.92 ROC in predicting long-term stays (more than 5 days). External validation using the SVM model demonstrated 75% ROC values. Factors such as fast-track protocols, anastomosis, and drainage emerged as key predictors of LoS. Integrating MLTs into predicting postoperative LOS in colon cancer surgery offers a promising avenue for personalized patient care and improved surgical management. Using intraoperative features in the algorithm enables the profiling of a patient’s stay based on the planned intervention. This issue is important for tailoring postoperative care to individual patients and for hospitals to effectively plan and manage long-term stays for more critical procedures.

## 1. Introduction

The surgical management of colon cancer, particularly the extent of lymphadenectomy and the impact of surgical techniques on postoperative outcomes, has been a subject of ongoing research and debate within the medical community [1]. Recent advancements in laparoscopic surgery, including the principles of complete mesocolic excision (CME) and central vascular ligation (CVL), have shown promise in improving oncological outcomes. The peculiarity of right hemicolectomy surgery for colon cancer, in Italy, has been explored in the CoDIG studies [2,3], underscoring surgeons’ commitment to advancing operative practices and patient care.

The CoDIG 1 study is a multicenter research project that has laid the groundwork for understanding the variability in surgical techniques and their impact on patient outcomes across different medical centers [3]. The CoDIG 2 study was based on the findings of the CoDIG 1 study and aimed to validate and extend its insights by focusing on the effectiveness of surgical techniques and the integration of new medical practices [2].

In this research framework, predicting and profiling the LoS in hospitals, especially after surgical procedures, is important for several reasons. It enables healthcare providers to optimize resource allocation, ensuring that beds, staff, and other essential resources are available and used efficiently [4]. By anticipating how long patients will stay, hospitals can better manage admissions and discharges, reduce bottlenecks, and improve the overall patient flow [5]. Moreover, accurately predicting LoS could help to identify patients at risk of prolonged stays, allowing healthcare teams to tailor postoperative care plans, implement targeted interventions early, and potentially shorten the duration of hospitalization [6,7].

In clinical research, the use of machine learning techniques (MLTs) for surgical outcome prediction represents a significant advancement over classical statistical models. MLT algorithms can process complex datasets with multiple variables, thereby capturing the nonlinear relationships and interactions that classical models may overlook [8].

Integrating MLTs into the predictive framework enhances the accuracy of postoperative hospital stay predictions, enabling personalized patient care and setting new standards in surgical research and practice [9]. This proactive approach facilitates efficient resource allocation and more effective interventions. The application of MLTs in this context aligns with the state of the art in medical research, pushing the boundaries of personalized medicine and setting new standards for surgical care [10].

Despite its potential benefits, the use of MLTs to predict the LoS after right hemicolectomy for colon cancer is currently underrepresented in the literature. This gap highlights the significant opportunity for research and development in surgical care.

This study aimed to consider MLTs to predict the postoperative LoS in patients undergoing right hemicolectomy for colon cancer using data from the CoDIG 1 and CoDIG 2 studies. This study aimed to fill a gap in the literature by providing an assessment of the predictive power of MLTs in this specific surgical context.

## 2. Materials and Methods

### 2.1. Data

The MLT models for predicting LoS were internally validated on the CoDIG 1 [3] dataset and externally validated on the CoDIG 2 [2] data.

- The CoDIG 1 [3] data were obtained from a large, multicenter, Italian study aimed at evaluating the surgical outcomes associated with two different techniques of ileocolic anastomosis (intracorporeal [ICA] and extracorporeal [ECA]) during laparoscopic right hemicolectomy. This prospective cohort study, endorsed by the Italian Society of Endoscopic Surgery and New Technologies (SICE), involved 85 surgical units across Italy, which contributed data on 1225 patients who underwent elective laparoscopic or robotic right hemicolectomy between March 2018 and September 2018.
- CoDIG 2 [2] data were used to externally validate the MLTs. The CoDIG 2 study is an observational multicenter national study involving 76 Italian surgical wards specializing in colorectal surgery aimed at assessing the practices of Italian surgeons regarding the extent of lymphadenectomy performed during right hemicolectomy (RH) for colon cancer. We sought to understand the current surgical approaches and any evolving trends compared with the previous CoDIG 1 study.

Other details of the aforementioned studies are reported in the literature [2,3].

### 2.2. Descriptive Statistics

Descriptive information about the data was conveyed by presenting the medians and interquartile ranges for quantitative variables and the absolute and relative frequencies for qualitative variables. The data were categorized based on the LoS, specifically highlighting stays longer than one week for descriptive purposes. To analyze the differences between groups, the Wilcoxon test was used for quantitative variables, whereas the chi-square test or, if suitable, the Fisher exact test was used for qualitative ones.

The multivariable logistic regression model OR, calculated on the training sample, with 95% confidence intervals on the risk of a prolonged stay (more than one week), is also reported for descriptive purposes in the Supplementary Material.

### 2.3. Machine Learning

#### 2.3.1. Patient Variables

This study focused on a subset of variables deemed relevant for predicting LoS, such as patient demographics, pre-existing comorbidities, the American Society of Anesthesiologists (ASA) scores, surgical details, and intraoperative variables. The variables used for training the models are summarized in Table 1 and described in Section S1.

#### 2.3.2. Machine Learning Model Training and Validation

To predict LoS, several MLTs have been employed, including random forest (RF) [11,12], gradient-boosting machine (GBM) [13], generalized linear model via penalization (GLMNET) [14], support vector machine (SVM) [15] with a linear separation function, and traditional linear models (LMs). Other details concerning the algorithms are reported in Supplementary Material Section S1.

The caret package [16] in R 4.3.2 [17] was used for model training. We applied 100 bootstrap runs with an optimism correction for model validation and internal training.

Each model was trained on CoDIG 1 [3] data using a set of predictors, dynamically selected by bootstrap resampling based on preprocessing outcomes, and evaluated using the mean absolute percentage error (MAPE) for regression tasks. Preprocessing steps, such as centering and scaling, were applied to optimize model performance.

The comparison across models focused on the MAPE for regression models to identify the best-performing algorithm. The proportion of correct predictions within an LoS of three days was also computed as a model performance measure with the root-mean-square error (RMSE). The ROC for the prediction score in identifying a prolonged stay (longer than 5.48 days) was reported. This quantity represented the average stay duration (4.24 days) plus the standard deviation (1.24) reported as performing weighted averages, for the study size, of the data reported in the recent review by Meyer and colleagues [18].

The model performance measures were also calculated to predict the LoS in the external validation set CoDIG 2 [2].

#### 2.3.3. Variable Importance

To assess the relative importance of the variables in our predictive models, we implemented a variable importance evaluation method tailored to measure the contribution of each predictor to the model’s predictive ability. This approach allowed us to identify the factors most influential in predicting the outcomes of interest, such as the length of hospital stay.

The SHAP (Shapley additive explanations) [19] importance was considered for assessing the variables’ importance. The SHAP values provided a measure of each variable’s contribution to the MLT’s predictions.

This metric assigned each variable a value that represented its importance in making a particular prediction, based on the concept of Shapley values from cooperative game theory.

The selected model was the best algorithm, satisfying a suitable performance in both the training and validation samples.

#### 2.3.4. Shiny Application Development

A Shiny application was developed to operationalize the best-performing MLT model for the real-time prediction of postoperative LoS in patients undergoing right hemicolectomy. This web-based tool was built with RStudio’s Shiny 1.8.0 [20] package, enabling healthcare professionals to input patient-specific data and receive immediate LoS estimates (see the Supplementary Materials).

## 3. Results

This machine was developed on a sample of 1224 patients who underwent colorectal surgery and were categorized into two groups according to the length of their postoperative hospitalization: individuals with a time of less than one week (*n* = 570) and those with a period longer than one week (*n* = 654).

One patient with no information on their postoperative LOS was excluded from the training. The machines were externally validated using 788 CoDIG 2 data (Table 1).

Table 1 presents the patients’ demographic and clinical characteristics used to train the models in both samples. The demographic variables, including age and sex, showed no statistically significant differences between the two groups, such as BMI categories, in the training sample (Table 1). Conversely, the presence of blood transfusion, type of anastomosis, implementation of drainage, conversion to open surgery, and adherence to fast-track protocols showed statistically significant differences between the groups. Elevated rates of blood transfusion, employment of extracorporeal anastomosis, postoperative drainage, conversion to open surgery, and non-compliance with fast-track protocols are all associated with prolonged hospital stays. Borderline results were observed for the type of right hemicolectomy and the length of the surgical procedure (Table 1).

In the CoDIG 2 dataset, instead, patients who remained in the hospital for more than one week were older, with a higher ASA score severity and comorbidities. Differences were also observed according to the tumor and node staging. Moreover, a longer hospital stay was associated with a longer surgical duration (Table 1). The median LoS was 7 (5–8) days in CoDIG 1 and 6 (5–8) in CoDIG 2.

To predict the length of hospital stay, we applied several MLTs (Table 2).

RF exhibited the lowest MAPE and RMSE and the highest accuracy among all the models in the training sample, indicating a remarkable predictive capability and overall precision. GBM presented a marginally higher MAPE than RF; however, the MAPE remained sufficiently low. Its RMSE was higher than that of RF but was still reasonably low. This may represent an acceptable choice for balancing the predictive accuracy and model complexity. SVM had a lower MAPE than GBM but it was still higher than that of RF. Such a value could be acceptable, but its RMSE was the highest among all the models. Finally, GLMNET and LM presented similar values for both metrics and were worse than those of RF.

Considering the external validation sample, the best-performing algorithm for all metrics was SVM, followed by the RF model (Table 1).

**Figure 1:**
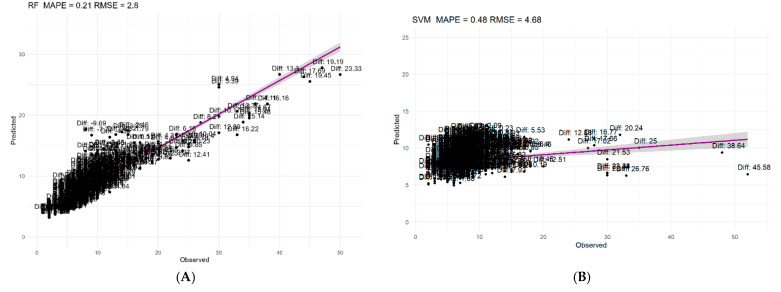
Observed versus predicted values of the RF model in training (**A**) and SVM (**B**) in test for LOS.

Figure 1 shows a plot of the predicted versus observed values for the RF and SVM best-performing algorithms used to visually assess the prediction ability and precision of the training sample. The figure confirms the superior performance of RF in predicting the outcomes of the training sample.

**Figure 2:**
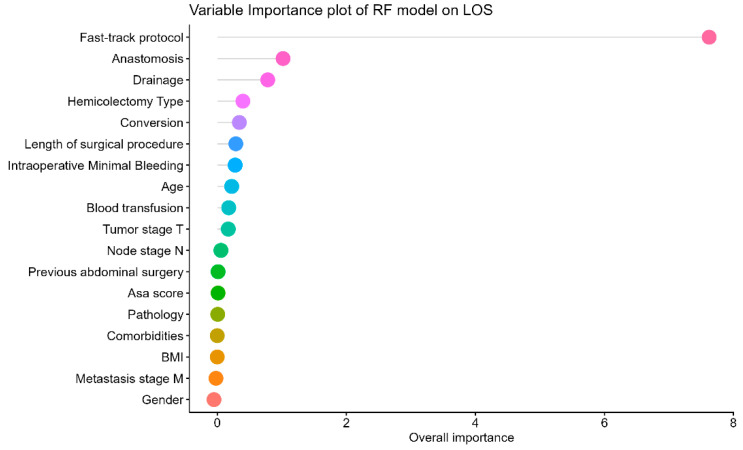
Variable importance plot for LOS with SVM algorithm. Anastomosis indicates the use of intracorporeal anastomosis, conversion indicates the conversion to open surgery, and hemicolectomy type indicates the surgical approach (open, laparoscopic, or video-assisted, where video-assisted surgery refers to a hybrid laparoscopic technique with anastomosis performed through service access).

Figure 2 shows the SVM’s variable importance plot (VIP), where all the variables are plotted with their importance measure. The leading LoS predictors identified by the VIP plot were the fast-track protocol, anastomosis, and drainage use in the surgical procedure. For comparison purposes, the same relevant significant variables were identified by considering a logistic regression model calculated based on the risk of a prolonged stay (more than one week). The fast-track protocol, anastomosis, and drainage revealed more precise effects with a shorter confidence interval length (Figure S1). The fast-track protocol with anastomosis and a short length of the surgical procedure showed a protective effect against prolonged hospital stay. Drainage, intraoperative minimal bleeding, and conversion were risk factors for a prolonged stay (Figure S1).

## 4. Discussion

The CoDIG [2,3] studies have contributed to the expanding body of evidence supporting the shift from conventional open surgery to laparoscopic techniques. This transition offers patient benefits, including a reduction in postoperative pain, a decrease in hospital stays, and a quicker resumption of normal activities [21,22]. These patient-centered outcomes are of utmost importance in the context of cancer surgery, where the quality of life post-surgery is a crucial oncological outcome [23].

The proposed research contributes to surgical oncology by predicting the LoS for most laparoscopic right hemicolectomy (RH) cases using the developed MLT model. This predictive tool could assist in better hospital resource management and set realistic recovery expectations for patients and families [4]. This is especially important in high-demand facilities where bed space and staff are limited. Forecasting long-term stays helps hospitals allocate staff, plan discharges, coordinate follow-up care, reduce bottlenecks, and enhance overall efficiency [24].

The superior performance of SVM over RF in external validation emphasizes the need for careful algorithm selection. SVM’s advantage suggests it may be less prone to overfitting, which affects the model performance on new data. This aligns with other studies showing RF and SVM as the top algorithms for LOS prediction [25]. This suggests that the SVM methodology for finding the optimal regression hyperplane might be better suited to the dataset’s characteristics in external validation scenarios, where the data may present different distributions or variable relationships than those observed in the training sample [26].

From a clinical standpoint, the identification of key variables in the VIP plot that influence the LoS underscores the significance of the fast-track protocol, anastomosis, and drainage as the primary predictors. The fast-track protocol, which includes elements like early mobilization, optimized pain management, and reduced perioperative fasting, has been shown to significantly shorten LoS and improve patient outcomes in colorectal surgery. This aligns with the enhanced recovery after surgery (ERAS) protocol literature [27], which consistently demonstrates that such measures lead to faster recovery, decreased complication rates, and enhanced patient satisfaction, thereby improving both clinical and operational efficiency [28,29]. Concerning the impact of adherence to the ERAS protocol, the literature evidence that, despite these recommendations, ERAS protocols are not widely adopted in Italy. One of the primary obstacles is the organizational difficulty in altering existing care pathways. Implementing an ERAS protocol requires significant resources and imposes considerable demands on the multidisciplinary team. Furthermore, a recent literature review found different perioperative management programs [30], each with considerable variations in their components and levels of compliance [31].

Anastomosis, the surgical connection between two structures, is another critical predictor. The type and quality of anastomosis can influence the recovery time and the likelihood of postoperative complications such as leaks, which are associated with longer hospital stays. Identifying its impact highlights the need for meticulous surgical techniques and postoperative monitoring to mitigate risks and enhance recovery [32]. Regarding the use of intracorporeal anastomosis, the data in the CoDIG 1 study demonstrated better short-term outcomes, including reduced hospital stays and postoperative pain [3]. These findings are further supported by a systematic review and meta-analysis, which confirm the benefits of intracorporeal anastomosis in improving recovery and minimizing postoperative discomfort [33].

Prophylactic abdominal drains have been historically used in colorectal surgery for removing harmful fluid collections and early detection of complications [34]. However, recent evidence indicates that drains may increase the risk of surgical site infections and adhesions [35]. This finding emphasizes the importance of postoperative care and the protocols surrounding the use of drains [36]. In our data, drainage use was associated with a prolonged hospital stay. Evidence suggests that drains can increase serous fluid production, risk of surgical site infection, and adhesions, and prolong hospital stays, impacting pain control, mobility, discomfort, and anxiety [35]. The ERAS Society, American Society of Colon and Rectal Surgeons, and other guidelines recommend against routine use of pelvic and peritoneal drains in colorectal surgery based on moderate-quality evidence from RCTs and meta-analyses [37]. However, drainage is still adopted in surgical practice, and many surgeons, especially in Europe and China, still believe prophylactic drainage reduces the risk of complications and aids the early detection of issues like intra-abdominal bleeding or anastomotic leakage [38].

Furthermore, factors such as conversion to open surgery, intraoperative minimal bleeding, and the length of the surgical procedure, while less impactful on the prediction algorithm’s performance, are significant predictors of prolonged stays in a logistic regression model, even if with higher variability in the effect. Conversion to open surgery increases the operative duration and blood loss, adversely affecting patient stay and post-surgical outcomes, as reported in the literature [39]. Minimally invasive techniques like laparoscopy reduce hemorrhage and the risks associated with perioperative transfusions, improving patient care [39].

Surgical outcomes also depend on surgeons’ experience and require tailored goals based on hospital-specific criteria. Successful ERAS implementation necessitates a well-informed, collaborative team with updated protocol knowledge and clearly defined roles. Continuous adaptation and post-implementation research are essential for refining protocol adherence. Other research efforts are useful in this direction [40].

Our study confirms that fast-track protocols significantly reduce LoS, supporting the literature on this topic. While fast-track implementation is important, it is not a preoperative factor that can predict LOS. Instead, our findings highlight the importance of using MLTs to identify patients at risk of prolonged stays based on preoperative and intraoperative data. This proactive approach not only improves patient recovery but also provides a stronger incentive for adopting these risk prediction models in clinical practice.

Moreover, the utilization of intraoperative variables in the algorithm enables the detailed profiling of a patient’s stay based on the planned intervention. This aspect, firstly, allows healthcare providers to tailor postoperative care plans more precisely for individual patients. By understanding the expected course of recovery, medical teams can anticipate potential complications, allocate appropriate resources, and ensure that each patient receives the necessary attention and interventions to promote optimal healing [41].

These results should encourage further exploration and refinement of machine learning models in the context of surgical outcomes. Future research could focus on integrating more diverse datasets, considering additional postoperative outcomes, and exploring the use of these models in real-time clinical decision support systems [42].

### Limitations

Our study recognizes several limitations. Firstly, the parameters influencing LoS, such as fast-track protocols, anastomosis type, and drain policy, are well-documented and largely dependent on the clinical practices and expertise within a department. Fast-track protocols require a departmental commitment to advanced training and horizontal integration among surgeons, nurses, theatre staff, anesthetists, and ward care. Similarly, the choice of anastomosis and drain policy relies heavily on the proficiency of highly trained surgeons.

Moreover, while our predictive models effectively identify patients at risk for prolonged stays using intraoperative and preoperative data, these factors cannot be preoperatively predicted. However, this limitation underscores its utility in proactive planning management and resource allocation.

Future research should focus on refining these models by incorporating more granular data on surgical practices and exploring additional preoperative predictors to enhance their applicability.

## 5. Conclusions

This study reports an MLT predictive tool for postoperative hospital stays in right hemicolectomy colon cancer surgery patients, utilizing data from the CoDIG studies. The relevant predictors of LoS identified underscore the significance of the fast-track protocol, anastomosis, and drainage as the primary predictors. The implementation of the predictive tool promises to improve healthcare delivery by enabling personalized patient care and optimizing resource allocation. This research paves the way for future advancements in patient-centered care, emphasizing the need for broader validation and exploration of MLTs in healthcare.

## Figures and Tables

**Table 1:** Baseline demographic and clinical characteristics of the CoDIG 1 training sample and CoDIG 2 external validation set.

	CoDIG 1 (Internal Validation Sample)				CoDIG 2 (External Validation Sample)			
Variables	Less than 1 Week	More than 1 Week	Total	*p*	Less than 1 Week	More than 1 Week	Total	*p*
	(*n* = 570)	(*n* = 654)	(*n* = 1224)		(*n* = 454)	(*n* = 334)	(*n* = 788)	
Preoperative								
Age (years) (median [IQR])	74.0 [65.0;80.0]	73.0 [64.0;80.0]	74.0 [65.0;80.0]	0.315	73.0 [65.0;80.0]	76.0 [70.0;82.0]	74.0 [67.0;81.0]	<0.001
Gender, n (%)				0.99				0.075
Male	293 (51.4%)	337 (51.5%)	630 (51.5%)		228 (50.2%)	190 (56.9%)	418 (53.0%)	
Female	277 (48.6%)	317 (48.5%)	594 (48.5%)		226 (49.8%)	144 (43.1%)	370 (47.0%)	
BMI, n (%)				0.137				0.421
<18	13 (2.28%)	27 (4.13%)	40 (3.27%)		10 (2.20%)	4 (1.20%)	14 (1.78%)	
18–24	272 (47.7%)	294 (45.0%)	566 (46.2%)		216 (47.6%)	154 (46.1%)	370 (47.0%)	
25–30	203 (35.6%)	253 (38.7%)	456 (37.3%)		175 (38.5%)	126 (37.7%)	301 (38.2%)	
>30	82 (14.4%)	80 (12.2%)	162 (13.2%)		53 (11.7%)	50 (15.0%)	103 (13.1%)	
ASA score, n (%)				0.2				<0.001
I	42 (7.37%)	51 (7.80%)	93 (7.60%)		23 (5.07%)	4 (1.20%)	27 (3.43%)	
II	269 (47.2%)	338 (51.7%)	607 (49.6%)		238 (52.4%)	121 (36.2%)	359 (45.6%)	
III	237 (41.6%)	250 (38.2%)	487 (39.8%)		179 (39.4%)	173 (51.8%)	352 (44.7%)	
IV	22 (3.86%)	15 (2.29%)	37 (3.02%)		14 (3.08%)	36 (10.8%)	50 (6.35%)	
Pathology, n (%)				0.562				0.314
Benign	77 (13.5%)	80 (12.2%)	157 (12.8%)		26 (5.73%)	13 (3.89%)	39 (4.95%)	
Malignant	493 (86.5%)	574 (87.8%)	1067 (87.2%)		428 (94.3%)	321 (96.1%)	749 (95.1%)	
Comorbidities, n (%)				0.987				0.001
None	330 (57.9%)	380 (58.1%)	710 (58.0%)		297 (65.4%)	180 (53.9%)	477 (60.5%)	
One or more	240 (42.1%)	274 (41.9%)	514 (42.0%)		157 (34.6%)	154 (46.1%)	311 (39.5%)	
Previous abdominal surgery, n (%)				0.635				0.297
None	299 (52.5%)	353 (54.0%)	652 (53.3%)		252 (55.5%)	172 (51.5%)	424 (53.8%)	
One or more	271 (47.5%)	301 (46.0%)	572 (46.7%)		202 (44.5%)	162 (48.5%)	364 (46.2%)	
Tumor, n(%)				0.255				<0.001
T1	69 (14.7%)	66 (12.3%)	135 (13.5%)		68 (17.0%)	33 (11.2%)	101 (14.6%)	
T2	94 (20.1%)	114 (21.3%)	208 (20.7%)		121 (30.3%)	57 (19.4%)	178 (25.7%)	
T3	249 (53.2%)	271 (50.7%)	520 (51.8%)		172 (43.1%)	161 (54.8%)	333 (48.1%)	
T4	56 (12.0%)	84 (15.7%)	140 (14.0%)		38 (9.52%)	43 (14.6%)	81 (11.7%)	
Node, n (%)				0.6				0.016
N+	58 (12.2%)	75 (13.9%)	133 (13.1%)		34 (8.59%)	45 (15.5%)	79 (11.5%)	
N0	308 (64.6%)	348 (64.7%)	656 (64.6%)		258 (65.2%)	169 (58.3%)	427 (62.2%)	
N1	111 (23.3%)	115 (21.4%)	226 (22.3%)		104 (26.3%)	76 (26.2%)	180 (26.2%)	
Metastasis, n(%)				0.496				0.187
M0	443 (94.7%)	498 (93.4%)	941 (94.0%)		377 (95.4%)	270 (92.8%)	647 (94.3%)	
M+	25 (5.34%)	35 (6.57%)	60 (5.99%)		18 (4.56%)	21 (7.22%)	39 (5.69%)	
Intraoperative								
Length of surgical procedure, n (%)				0.089				<0.001
>270 min	27 (4.74%)	48 (7.34%)	75 (6.13%)		37 (8.15%)	46 (13.8%)	83 (10.5%)	
181–270 min	180 (31.6%)	220 (33.6%)	400 (32.7%)		152 (33.5%)	141 (42.2%)	293 (37.2%)	
90–180 min	363 (63.7%)	386 (59.0%)	749 (61.2%)		265 (58.4%)	147 (44.0%)	412 (52.3%)	
Blood transfusion, n (%)				<0.001				<0.001
No	20 (3.51%)	59 (9.02%)	79 (6.45%)		448 (98.7%)	312 (93.4%)	760 (96.4%)	
Yes	550 (96.5%)	595 (91.0%)	1145 (93.5%)		6 (1.32%)	22 (6.59%)	28 (3.55%)	
Intraoperative minimal bleeding > 200 mL, n (%)				<0.001				0.001
No	551 (96.7%)	603 (92.2%)	1154 (94.3%)		419 (92.3%)	288 (86.2%)	707 (89.7%)	
Yes	19 (3.33%)	51 (7.80%)	70 (5.72%)		35 (7.71%)	46 (13.8%)	81 (10.3%)	
Anastomosis, n (%)				<0.001				<0.001
Extracorporeal	87 (15.3%)	276 (42.2%)	363 (29.7%)		89 (19.6%)	121 (36.2%)	210 (26.6%)	
Intracorporeal	483 (84.7%)	378 (57.8%)	861 (70.3%)		365 (80.4%)	213 (63.8%)	578 (73.4%)	
Drainage, n(%)				<0.001				<0.001
No	303 (53.2%)	171 (26.1%)	474 (38.7%)		185 (40.7%)	92 (27.5%)	277 (35.2%)	
Yes	267 (46.8%)	483 (73.9%)	750 (61.3%)		269 (59.3%)	242 (72.5%)	511 (64.8%)	
Conversion *, n (%)				<0.001				0.004
No	566 (99.3%)	592 (90.5%)	1158 (94.6%)		431 (94.9%)	298 (89.2%)	729 (92.5%)	
Yes	4 (0.70%)	62 (9.48%)	66 (5.39%)		23 (5.07%)	36 (10.8%)	59 (7.49%)	
Fast-track protocol, n (%)				<0.001				<0.001
No	135 (23.7%)	435 (66.5%)	570 (46.6%)		80 (17.6%)	167 (50.0%)	247 (31.3%)	
Yes	435 (76.3%)	219 (33.5%)	654 (53.4%)		374 (82.4%)	167 (50.0%)	541 (68.7%)	
Right hemicolectomy **, n(%)				0.068				0.262
Laparoscopic	513 (90.0%)	575 (87.9%)	1088 (88.9%)		369 (81.3%)	278 (83.2%)	647 (82.1%)	
Robotic	34 (5.96%)	60 (9.17%)	94 (7.68%)		59 (13.0%)	32 (9.58%)	91 (11.5%)	
Video-assisted ***	23 (4.04%)	19 (2.91%)	42 (3.43%)		26 (5.73%)	24 (7.19%)	50 (6.35%)	
Outcome								
LoS	3.00 [2.00;3.00]	7.00 [5.00;9.00]	7.00 [5.00;8.00]	<0.001	6.00 [5.00;8.00]	8.00 [7.00;11.0]	6.00 [5.00;8.00]	<0.001

* Conversion indicates conversion to open surgery; ** hemicolectomy type indicates the surgical approach (open, laparoscopic, or video-assisted); *** video-assisted surgery refers to a hybrid laparoscopic technique with anastomosis performed through service access.

**Table 2:** Comparison of model performance for LOS. Internal validation (CoDIG 1) measures were reported together with external validation measures (CoDIG 1). MAPE, RMSE, and accuracy were defined as the proportion of records correctly classified within three days. The ROC is the ability of the algorithm to predict a long-term stay (>5 days). The best-performing algorithms have been highlighted in bold text.

	CoDIG 1 Internal Validation				CoDIG 2 External Validation			
Model	MAPE	RMSE	ROC	Accuracy	MAPE	RMSE	ROC	Accuracy
Random forest (RF)	**0.21**	**2.8**	**0.92**	**0.94**	0.81	6.04	0.65	0.42
Support vector machine (SVM)	0.29	5.00	0.86	0.83	**0.48**	**4.68**	**0.75**	**0.79**
Gradient-boosting machine (GBM)	0.38	4.78	0.81	0.81	0.91	6.15	0.67	0.3
Generalized linear model with penalized maximum likelihood (GLMNET)	0.38	4.8	0.8	0.81	0.76	5.54	0.68	0.43
Linear model (LM)	0.38	4.75	0.78	0.8	0.93	6.3	0.67	0.3

## Data Availability

Data are available upon reasonable request to the corresponding author.

## Supplementary Materials

The following supporting information can be downloaded at https://www.mdpi.com/article/10.3390/cancers16162857/s1, Figure S1: Panel A: Multivariable Logistic Regression Odds Ratio (OR) with 95% Confidence Intervals (CI). Panel B Log OR Plot with 95% CI; Figure S2: web app interface.

## Abbreviations

CME	Complete mesocolic excision
CVL	Central vascular ligation
LoS	Length of stay
MLT	Machine learning techniques
RF	Random forest
RMSE	Root-mean-square error
SVM	Support vector machine
CME	Complete mesocolic excision
CVL	Central vascular ligation
SICE	Italian Society of Endoscopic Surgery and New Technologies
RH	Right hemicolectomy
ASA	American Society of Anesthesiologists
GBM	Gradient-boosting machine
GLMNET	Generalized linear model via penalization
SVM	Support vector machine
LM	Linear model
MAPE	Mean absolute percentage error
MSE	Mean square error
BMI	Body mass index
AUC	Area under curve
VIP	Variable importance plot
MDA	Mean decrease accuracy
RH	Right hemicolectomy
ERAS	Enhanced recovery after surgery
