# Supplementary material for: Predicting Postoperative Length of Stay in Patients Undergoing Laparoscopic Right Hemicolectomy for Colon Cancer: A Machine Learning Approach Using SICE (Società Italiana di Chirurgia Endoscopica) CoDIG Data
Source: Cancers (Basel). 2024 Aug 16;16(16):2857. doi: 10.3390/cancers16162857 (PMC11352329; doi:10.3390/cancers16162857)
Supplement: Supplementary file 1 [file cancers-16-02857-s001.zip › cancers-3117144-supplementary.pdf]

Section S1. ML Models

Classification and Regression Trees (CART) [1] are methods for constructing prediction models obtained by recursively partitioning the data space and fitting a simple prediction model within each partition. As a result, the partitioning can be represented graphically as a decision tree.

Random Forest (RF)[2] is a typical ML technique that recursively creates multiple decision trees. It selects a subset of available features and recursively partitions the data in the regression space until the amount of variation in the subspace is small. Random forest is a greedy technique and as a result, it does not necessarily converge to the global optimal solution. To avoid such indecisive convergence, a collection or ensemble of locally optimal trees can be done (bagging.) The ensemble of such trees is known as a forest.

***Linear Model (LM)* with elastic net regularization** [3] is a regularized regression method that linearly combines the L1 and L2 penalties of the lasso and ridge methods applied in sinergy with a link function and a variance function to overcome linear model limitation (such as the constant variability among the mean and the normality of the data). A **simple Linear (LM)** Regression model has been also considered.

The Support Vector Machine (SVM) algorithm's main objective is to find an optimal hyperplane of the feature's N-dimensional space (N—the number of variables) that distinctly classifies the data points into a binary partition. Several hyperplanes may separate the classes of data points. The SVM algorithm considers the hyperplanes, which maximize the margin (the distance between data points of classes). The functional form of Hyperplane separators may be **linear** or smoothed via the **Kernel** density function. Both parameterizations have been considered[4].

Gradient Boosting is a ML technique that sequentially builds predictive models. It begins with a base model and incrementally improves its predictions by correcting errors from previous stages. This approach combines multiple weak predictive models, typically decision trees, to create a single strong model. Each new model focuses on accurately predicting the residuals or errors of the prior models, enhancing the overall prediction accuracy with each step[5].

Variables considered for training the models

- Age (years);
- Gender;
- BMI, Body Mass Index categorized as:
    - <18: Underweight patients.
    - 18-24: Normal weight patients.
    - 25-30: Overweight patients.
    - >30: Obese patients.
- ASA score. The American Society of Anesthesiologists (ASA) score classifies patients' preoperative physical status:
    - I: Healthy patients.
    - II: Patients with mild systemic disease.
    - III: Patients with severe systemic disease.
    - IV: Patients with severe systemic disease that is a constant threat to life.
- Pathology. This variable distinguishes between:

    - Benign: Non-cancerous conditions.
    - Malignant: Cancerous conditions.
- Comorbidities, any;
- Previous abdominal surgery, shows whether patients have had:
    - None: No prior abdominal surgeries.
    - One or more: One or more previous abdominal surgeries.
- Tumor staging is categorized as:
    - T1: Small, localized tumors.
    - T2: Larger tumors that have not spread.
    - T3: Tumors that have spread to nearby tissues.
    - T4: Tumors that have spread to other organs or structures.
- Metastasis, indicates whether patients have:
    - M0: No distant metastasis.
    - M+: Presence of distant metastasis.

Intraoperative

- Length of surgical procedure, categorized as:
    - >270 min: Surgeries longer than 270 minutes.
    - 181-270 min: Surgeries lasting between 181 and 270 minutes.
    - 90-180 min: Surgeries lasting between 90 and 180 minutes.
- Blood Transfusion, Indicates whether patients require a blood transfusion:
- Intraoperative Minimal Bleeding (>200 ml), shows whether minimal bleeding occurred during surgery, according to the judgment of the clinician.
- Anastomosis, a type of surgical connection between structures:
    - Extracorporeal: Performed outside the body.
    - Intracorporeal: Performed inside the body.
- Drainage, indicates the use of surgical drainage:
- Conversion, shows whether the procedure was converted from minimally invasive to open surgery[;
- Fast-track protocol, indicates whether a fast-track recovery protocol was used;
- Right hemicolectomy, indicates the type of surgical approach for right hemicolectomy:
    - Laparoscopic: Minimally invasive laparoscopic surgery.
    - Robotic: Robotic-assisted surgery.
    - Video-assisted: Video-assisted surgery.

Figure S1 Panel A: Multivariable Logistic Regression Odds Ratio (OR) with 95% Confidence Intervals (CI). Panel B Log OR Plot with 95% CI.

Panel A Odds Ratios with Confidence Intervals

Conversion	4.64 (1.69, 16.52)
Intraoperative Minimal Bleeding >200 ml	3.25 (1.59, 6.89)
Drainage	1.72 (1.24, 2.37)
Tumor stage T4	1.63 (0.87, 3.07)
Asa score III	1.51 (0.71, 3.21)
Asa score II	1.50 (0.76, 2.99)
Robotic, Hemicolecomy Type	1.44 (0.84, 2.48)
Tumor stage T2	1.32 (0.78, 2.24)
Comorbidities	1.32 (0.91, 1.90)
Tumor stage T3	1.26 (0.78, 2.05)
Pathology malignant	1.15 (0.33, 4.14)
Node stage N0	1.02 (0.61, 1.71)
Age	0.99 (0.98, 1.01)
BMI	0.96 (0.78, 1.18)
Asa score IV	0.93 (0.29, 2.91)
Previous abdominal surgery	0.92 (0.68, 1.25)
Node stage N1	0.88 (0.51, 1.51)
Gender M	0.83 (0.61, 1.13)
Metastasis stage M0	0.73 (0.36, 1.45)
Blood transfusion	0.57 (0.28, 1.11)
Video Assisted, Hemicolecomy Type	0.56 (0.23, 1.29)
Length of surgical procedure 90-180	0.55 (0.28, 1.08)
Anastomosis Intracorporeal	0.43 (0.30, 0.63)
Length of surgical procedure 181-270	0.41 (0.20, 0.82)
Fast-track protocol	0.20 (0.15, 0.27)

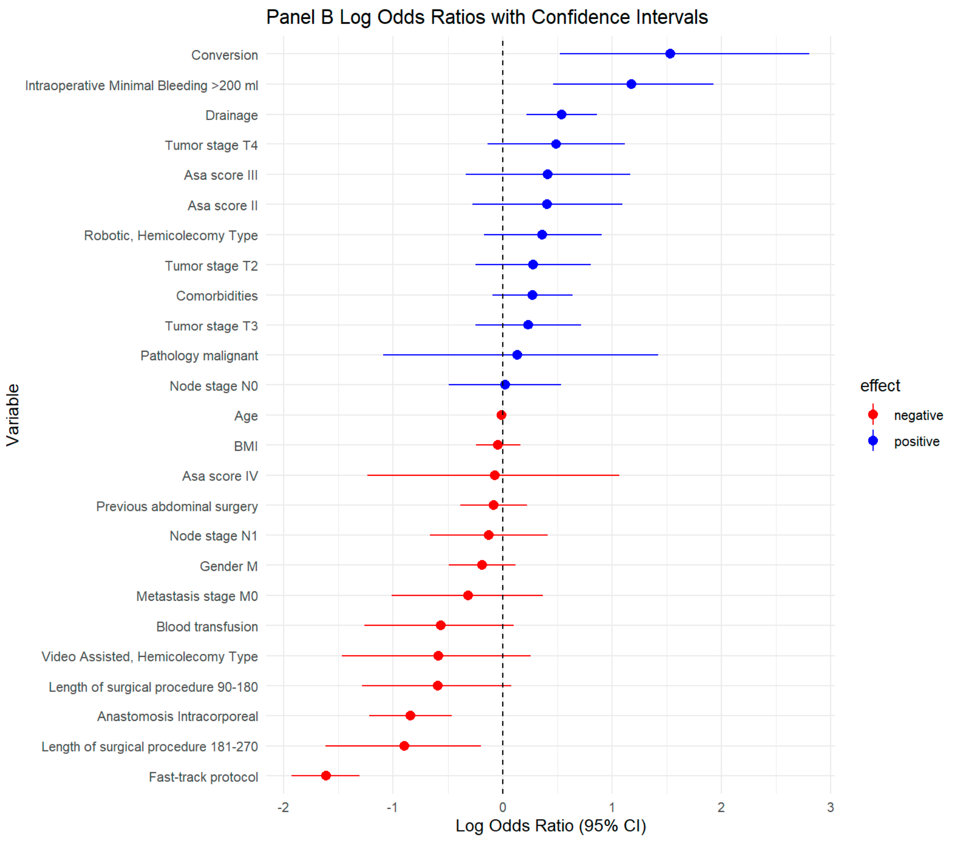

Conversion is the conversion to Open surgery, and the Hemicolectomy type indicates the surgical approach. Video-assisted surgery refers to a hybrid laparoscopic technique with anastomosis performed through service access).

Description of the Shiny App

The provided Shiny app (https://biostatlab.shinyapps.io/APP_mlt_codig/) is an interactive tool designed to for predicting the length of hospital stay (LOS) in colorectal surgery patients.

Figure S2 web app interface

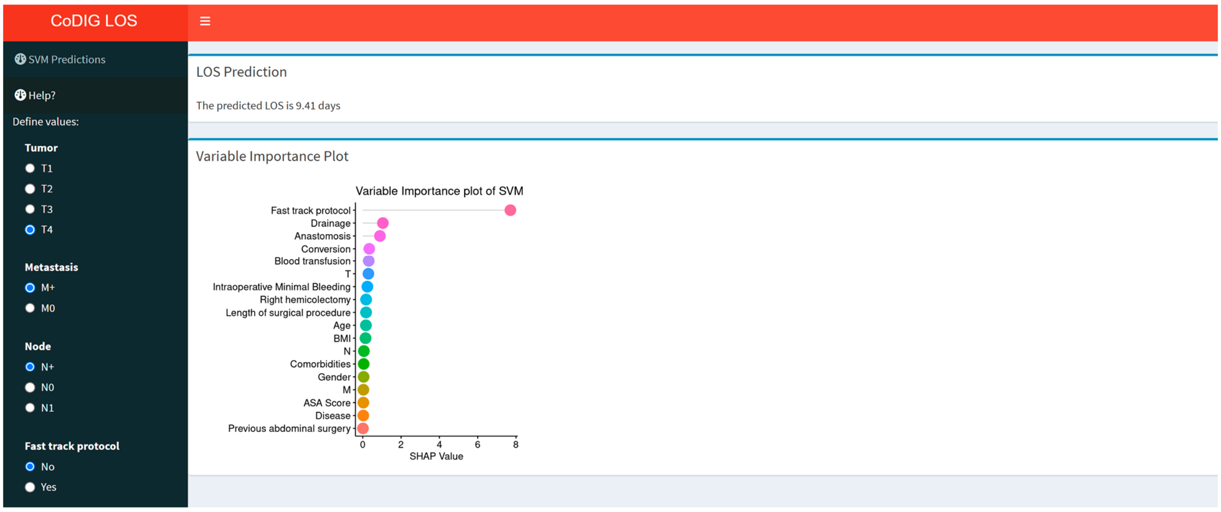

1. **Data entering**:
    - Users can insert patient data to see how the models predict LOS. The app processes the uploaded data and generates real-time predictions, providing instant feedback.
2. **Feature Importance**:
    - The app includes analyses of feature importance for each model, helping users understand which variables most influence the predictions. Feature importance is visualized using bar charts and other graphical tools.
3. **User-Friendly Interface**:
    - The app is designed with an intuitive and user-friendly interface, making it accessible to users with varying levels of expertise in machine learning and data analysis.

References

1. Breiman, L., Friedman, J. H., Olshen, R. A. & Stone, C. J. *Classification and Regression Trees*. (Wadsworth and Brooks/Cole, 1984).
2. Breiman, L. Random forests. *Machine Learning* **45**, 5–32 (2001).
3. Friedman, J., Hastie, T. & Tibshirani, R. Regularization Paths for Generalized Linear Models via Coordinate Descent. *J Stat Softw* **33**, 1–22 (2010).

4. Cortes, C. & Vapnik, V. Support-vector networks. *Machine Learning* **20**, 273–297 (1995).

5. Friedman, J. H. Stochastic gradient boosting. *Computational Statistics & Data Analysis* **38**, 367–378 (2002).
